# Expression of concern: Long non-coding RNA CRNDE promote the progression of tongue squamous cell carcinoma through regulating the PI3K/AKT/mTOR signaling pathway

**DOI:** 10.1039/d2ra90007f

**Published:** 2022-02-09

**Authors:** Laura Fisher

**Affiliations:** Royal Society of Chemistry, Thomas Graham House Science Park, Milton Road Cambridge CB4 0WF UK advances-rsc@rsc.org

## Abstract

Expression of concern for ‘Long non-coding RNA CRNDE promote the progression of tongue squamous cell carcinoma through regulating the PI3K/AKT/mTOR signaling pathway’ by Zhongheng Yang *et al.*, *RSC Adv.*, 2019, **9**, 21381–21390. DOI: 10.1039/C9RA01321K.

*RSC Advances* is publishing this expression of concern in order to alert readers that concerns have been raised over the integrity of the data published in this article. The authors have been contacted but have not responded to requests to provide raw data. An expression of concern will continue to be associated with the article until a conclusive outcome is reached.

Laura Fisher

31st January 2022

Executive Editor, *RSC Advances*

## Supplementary Material

